# The BRAVO Clinical Study Protocol: Oral Varespladib for Inhibition of Secretory Phospholipase A2 in the Treatment of Snakebite Envenoming

**DOI:** 10.3390/toxins15010022

**Published:** 2022-12-28

**Authors:** Rebecca W. Carter, Charles J. Gerardo, Stephen P. Samuel, Surendra Kumar, Suneetha D. Kotehal, Partha P. Mukherjee, Farshad M. Shirazi, Peter D. Akpunonu, Chanaveerappa Bammigatti, Ashish Bhalla, Neeraj Manikath, Timothy F. Platts-Mills, Matthew R. Lewin

**Affiliations:** 1 Ophirex, Inc., Corte Madera, CA 94295, USA; 2 Department of Emergency Medicine, Duke University, Durham, NC 27708, USA; 3 Department of Medicine, Sardar Patel Medical College, PBM Hospital, Bikaner 334001, India; 4 Department of Medicine, Mysore Medical College and Research Institute, Mysore 570001, India; 5 Department of General Medicine, Calcutta National Medical College, Kolkata 700014, India; 6 Arizona Poison & Drug Information Center, College of Pharmacy and University of Arizona College of Medicine, University of Arizona, Tucson, AZ 85721, USA; 7 Department of Emergency Medicine and Medical Toxicology, University of Kentucky College of Medicine, Lexington, KY 40506, USA; 8 Department of Medicine, Jawaharlal Institute of Postgraduate Medical Education and Research, Puducherry 605006, India; 9 Department of Internal Medicine, Postgraduate Institute of Medical Education and Research, Chandigarh 160012, India; 10 Department of Emergency Medicine, Government Medical College, Kozhikode 673008, India

**Keywords:** snakebite, clinical trial, protocol, BRAVO, varespladib, snakebite severity score, direct toxin inhibitor

## Abstract

Introduction: Snakebite is an urgent, unmet global medical need causing significant morbidity and mortality worldwide. Varespladib is a potent inhibitor of venom secretory phospholipase A_2_ (sPLA_2_) that can be administered orally via its prodrug, varespladib-methyl. Extensive preclinical data support clinical evaluation of varespladib as a treatment for snakebite envenoming (SBE). The protocol reported here was designed to evaluate varespladib-methyl for SBE from any snake species in multiple geographies. Methods and Analysis: BRAVO (Broad-spectrum Rapid Antidote: Varespladib Oral for snakebite) is a multicenter, randomized, double-blind, placebo-controlled, phase 2 study to evaluate the safety, tolerability, and efficacy of oral varespladib-methyl plus standard of care (SoC) vs. SoC plus placebo in patients presenting with acute SBE by any venomous snake species. Male and female patients 5 years of age and older who meet eligibility criteria will be randomly assigned 1:1 to varespladib-methyl or placebo. The primary outcome is the Snakebite Severity Score (SSS) that has been modified for international use. This composite outcome is based on the sum of the pulmonary, cardiovascular, nervous, hematologic, and renal systems components of the updated SSS. Ethics and Dissemination: This protocol was submitted to regulatory authorities in India and the US. A Clinical Trial No Objection Certificate from the India Central Drugs Standard Control Organisation, Drug Controller General-India, and a Notice to Proceed from the US Food and Drug Administration have been obtained. The study protocol was approved by properly constituted, valid institutional review boards or ethics committees at each study site. This study is being conducted in compliance with the April 1996 ICH Guidance for Industry GCP E6, the Integrated Addendum to ICH E6 (R2) of November 2016, and the applicable regulations of the country in which the study is conducted. The trial is registered on Clinical trials.gov, NCT#04996264 and Clinical Trials Registry-India, 2021/07/045079 000062.

## 1. Strengths and Limitations of this Study

Snakebite is an urgent, unmet, global medical need. BRAVO is the first clinical evaluation of a small-molecule direct toxin inhibitor (DTI) with the potential to provide broad-spectrum, safe, and cost-effective treatment in conjunction with the current standard of care.

To our knowledge, this protocol describes the first clinical trial for snakebite envenoming agnostic to snake species to be conducted in multiple geographies. The protocol was collaboratively designed to accommodate differing treatment guidelines, including a primary outcome intended for international use, assessment of envenoming using the 20-min whole blood clotting test (20WBCT), and simultaneous administration of standard of care to all participants.

The BRAVO trial design may provide a template for future international trials of novel treatments for SBE that allows for direct comparison between clinical trial results independent of intervention, SBE syndrome or geography.

One potential limitation of the current study is that the selection of specific time points for the primary endpoint might overlook or overemphasize organ systems that recover or manifest injury at different rates (e.g., neurotoxicity may change rapidly while tissue injury often recovers more slowly). Secondary endpoints that capture recovery over time are included to explore differential system recovery times.

## 2. Introduction

The treatment of snakebite envenoming (SBE) is an urgent, global unmet medical need. It is estimated that worldwide more than 2.7 million SBEs occur annually, causing significant morbidity (~400,000 permanent deformities or amputations) and mortality (~138,000 deaths), primarily among poor, rural populations of low- and middle-income countries [1,2,3]. Approximately 5.8 billion people live in regions that place them at risk for being bitten by medically important snakes. An estimated 75 percent of deaths from SBE occur outside the hospital setting before victims can reach adequate medical care [3,4] and poor outcomes are associated with delays in care [5,6,7]. A staggering 1.2 million snakebite deaths, 325,000 (28%) in children younger than 15 years of age, and loss of more than 3 million disability-adjusted life years are estimated to have occurred in India alone from 2000 to 2019 [8]. Although mortality is uncommon in the U.S., approximately 11,000 venomous snakebites occur annually with an estimated 1800 patients suffering severe tissue injury or significant hemotoxicity [9].

Antivenoms remain the cornerstone of SBE treatment, yet the global SBE epidemic remains intractable more than 120 years after their introduction [10]. The persistence of a high burden of morbidity and mortality results from therapeutic limitations as well as inadequate access. Antivenoms are highly specific, limiting their efficacy in treating SBE to the species (or closely related species) whose venoms were used in their production. Additionally, while antivenoms have proved to be effective in treating some of the lethal and damaging effects of SBE, they have limited efficacy with established neurotoxicity and local tissue necrosis, especially if not given early after envenoming. The limited efficacy of antivenoms for these conditions is partly a result of the poor tissue penetration of the antibodies that comprise antivenom [2]. Further, some of the key toxins in snake venom have low immunogenicity and so are not effectively neutralized by normal titers of antivenoms [11,12,13]. The requirement for intravenous administration as well as the anaphylactic and pyrogenic reactions associated with antivenom generally limit its use to hospital settings. Thus, species and regional specificity, complexity of safe administration, manufacturing complexity and expense, and storage and supply chain challenges all contribute to the need for a new therapeutic paradigm for SBE. Synthetic, direct toxin inhibitors (DTIs) of key, nearly ubiquitous toxins in snake venoms are at the forefront of new developments for snakebite therapies. In conjunction with the existing standard of care, toxin-specific small molecule therapies with broad species coverage, ease of safe administration, and inexpensive production have the potential to meaningfully reduce the global burden of SBE [14].

Secretory phospholipase A_2_ (sPLA_2_) enzymes represent a family of enzymes that hydrolyze phospholipids at the sn-2 position of the glycerol backbone. Several important SBE syndromes may mediated by sPLA_2_, one of the most pharmacologically active venom components present in 95% of venomous snake species [15,16]. It is thought to be one of the components responsible for lethality from presynaptic neurotoxins and long-term morbidity such as that affecting return to work and activities of daily living [2,17]. A variety of snake venom sPLA_2_ toxins lead to a heterogenous group of clinical syndromes including neurotoxicity, hemotoxicity, myotoxicity, hepatotoxicity, and nephrotoxicity [2,18,19,20]. Thus, inhibition of sPLA_2_ could address several limitations of antivenom therapies that lead to adverse outcomes, including the ability to reverse neurological effects not effectively treated by antivenom and to normalize coagulation parameters and platelet dysfunction that result from sPLA_2_ toxins. Nevertheless, it would be unwise to underestimate the complex interactions with other toxins: particularly those causing hemotoxicity/coagulopathy and skin-muscle injuries such as SVMPs, SVSPs, C-type lectin-like protein and 3FTx such that combination therapies are a likely evolution of using direct toxin inhibitors such as the sPLA2 inhibitor, varespladib [2,21,22].

Varespladib is a highly potent small molecule inhibitor of sPLA_2_ which along with its prodrug, varespladib-methyl has been tested in previous clinical trials in over 4600 patients for indications other than SBE, providing important information about their safety and pharmacokinetics [23]. Due to their low molecular weight (<400 g/mole) and other properties, varespladib can penetrate tissues not accessible to conventional antivenoms [23]. In vitro, varespladib potently inhibits snake venom sPLA_2_, with half-maximal inhibitory constant (IC_50_) values in the nano- and sub-nanomolar range for sPLA_2_ enzymes from vipers, pit vipers and elapids from around the world including Russell’s and saw-scaled vipers, copperheads, rattlesnakes, cobras, krait and taipan, among others [21]. The structural basis for this inhibition of snake venom sPLA_2_ has recently been established through crystallographic studies [24,25]. Following in vitro studies, the ability of varespladib to counteract sPLA_2_-dependent venom toxicity has been studied extensively in human blood as well as in mouse, rat, and pig animal models [21,26,27,28,29,30,31,32,33].

Extensive preclinical data support the hypothesis that varespladib is a broad-spectrum, DTI of sPLA2 that could help overcome some of the limitations of conventional antivenom [34]. We describe here a novel protocol for the first international clinical trial of a venom-agnostic treatment for SBE. As neutralization effect of antivenom is dependent on matched immunogenicity with specific snake venoms, previous clinical trials were designed to determine the efficacy of an antivenom against specific snake species or groups, limiting their geographic range. This protocol is the first clinical trial of a treatment for snakebite to be conducted in multiple geographies and against envenoming from any snake species. The protocol also seeks to broaden the approach to endpoints by capturing a range of patient-reported outcomes and clinician assessments covering common SBE syndromes. This randomized, double-blinded, placebo-controlled trial evaluates the safety, efficacy, and tolerability of a multi-dose regimen of oral varespladib with standard of care (SoC) in patients with SBE from a wide number of snake species, specifically those found in India and the U.S.

## 3. Methods

### 3.1. Study Design

This is a multicenter, randomized, double-blind, placebo-controlled, phase 2 study designed to evaluate the safety, tolerability and efficacy of oral varespladib-methyl plus SoC vs. SoC plus placebo in patients presenting with acute SBE. SPIRIT reporting guidelines were applied to trial design, Supplemental Table S4 [35]. Regardless of study group assignment, all patients will receive SoC, including antivenom, for snakebite as directed by the treating physician and in compliance with local, state, and national guidelines.

### 3.2. Setting

To adequately evaluate varespladib-methyl in a broad spectrum of potential snake species, patients will be enrolled at sixteen geographically diverse centers across the U.S. and India beginning in August 2021. Endemic venomous snakes at these sites include Viperidae, Viperinae, Crotalinae, and Elapidae families and sub-families. Participating clinical trial sites have reported treating bites from numerous species across several genera, including *Agkistrodon*, *Bungarus*, *Crotalus*, *Daboia*, *Echis*, *Hypnale*, *Micrurus*, *Naja*, and *Sistrurus* among others.

### 3.3. Participant Population

This study will enroll male and female patients 5 years of age and older who meet eligibility criteria summarized in Table 1. To be enrolled, subjects must present with sufficient signs and symptoms of snakebite as scored by the Snakebite Severity Score (SSS) inclusion categories: local wound, pulmonary, cardiovascular, hematologic, and nervous system (Table 2). Subjects completing the initial dose of antivenom must also have a Clinical Global Impression-Improvement (CGI-I) grade of minimally worse, much worse, or very much worse [36]. Point of care testing (e.g., 20WBCT) may be used for enrollment scoring in the Hematologic system. Point of care testing is only used for inclusion in the study. Confirmatory laboratory samples are collected concurrently with 20WBCT and are used for primary and secondary efficacy analyses. Patients with a history of acute coronary syndrome will be excluded [37,38,39].

### 3.4. Stratified Randomization and Blinding

Enrolled patients are assigned in a 1:1 ratio to varespladib-methyl or placebo using computer-generated randomization numbers assigned sequentially as patients are entered into the study. Enrollment is stratified by age group (a. 5 to <11 years, b. 11 to <18 years, and c. ≥18 years) and by the presence or absence of neurotoxicity at baseline, resulting in six strata in total.

All patients, investigators, and study personnel involved in the conduct of the study are blinded to treatment assignment. Individual patient unblinding may occur if required for medical emergency.

### 3.5. Intervention

Varespladib-methyl or placebo is administered concurrently with institutional SoC over a 7-day course of treatment. All investigational products, active and placebo, were manufactured and packaged under Good Manufacturing Practice conditions by Alcami Carolinas Corporation. The pharmacokinetic half-life of varespladib is approximately 10 h, thus dosing is administered twice a day (BID) to maintain adequate plasma concentrations. Adult patients receive an initial loading dose of 500 mg, followed by BID dosing with 250 mg for the remainder of the 7-day treatment period. Pediatric subjects ages 5 to 10 years are administered an initial dose of 200 mg then 100 mg; subjects ages 11 to 17 years are administered 400 mg then 200 mg. The study drug may be administered via feeding tube (e.g., naso- or orogastric tubes) in patients requiring mechanical ventilation. Investigators may discontinue study intervention if deemed participation is not in the best interest of the patient.

### 3.6. Study Procedures and Assessments

An abbreviated schedule of assessments is shown in Table 3. (full schedule of events provided in Supplementary Data, Table S2). Patient assessments will be performed by trained clinical research coordinators and investigators.

The primary outcome assessment of the BRAVO protocol is a version of the Snakebite Severity Score (SSS) that has been modified with the intention of making it more appropriate for international use. The original SSS is validated in previous clinical studies and is a widely accepted snakebite severity outcome measure based on six body system domains: local wound, pulmonary, cardiovascular, gastrointestinal, hematologic, and nervous system, Supplemental Table S3. Scores are based on a combination of symptoms, physical examination findings including vital signs, and laboratory values.

For this study, the SSS was modified for greater applicability to international use. A renal subscore was added at the recommendation of the India’s Subject Expert Committee to better characterize the frequency and burden of acute kidney injury following snakebite [40]. The newly added renal subscore was based on a validated international standard for grading kidney disease [41]. Baseline values for serum creatinine are defined as the most recent serum creatinine prior to envenoming, if one is available, or as the normal expected value for the patient. The modified SSS also includes death, severe weakness or paralysis and thromboses.

The primary endpoint for the trial is based on the sum of five components of the modified SSS: pulmonary, cardiovascular, nervous, hematologic, and renal systems. The primary endpoint does not include gastrointestinal or local wound systems as these are unlikely to be clinically relevant for the primary endpoint at 6 and 9 h post randomization. Local wound is a clinically relevant measure for chronic outcomes but is not always a reliable measure of acute outcomes. The extent of pain and bruising is difficult to measure objectively, and assessment is affected by bite depth variability and density of subcutaneous tissues as well as by some pre-hospital practices such as tourniquets and incisions. In contrast, SSS subcategories included in the primary endpoint (respiration, cardiovascular, hematological, neurological, and renal) are measurable and reflective of the underlying processes by which snakebite envenomings are life-threatening. All SSS domains, including gastrointestinal and local wound, are collected at each visit.

### 3.7. Outcome Measures

The primary and secondary outcomes are listed in Table 4. Safety and tolerability outcomes are listed in Table 5. The primary efficacy endpoint will be assessed by comparing the change in the combined pulmonary, cardiovascular, hematologic, renal, and nervous system sub-scores of the SSS from baseline (pre-dosing) to the average of the scores 6- and 9-h post-baseline for the treatment and control groups. Secondary efficacy endpoints are designed to describe SSS outcomes over the full treatment period and toxicity-specific and performance-related outcome measures. Additional patient-reported outcomes include the patient-specific functional scale, the numeric pain rating scale, and the patient global impression of change. An independent Data Safety Monitoring Board will meet at predetermined intervals to evaluate unblinded safety data.

### 3.8. Data Management

All study data will be de-identified and entered into an electronic database by study sites. Site visits will be conducted to monitor the study and ensure compliance with the protocol, GCP, and applicable regulations and guidelines.

### 3.9. Statistics

Descriptive statistical methods will be used to summarize the data from this study with confidence intervals calculated for the primary and secondary efficacy endpoints. *p*-values of less than 0.05 will be considered statistically significant based on a two-sided test. All statistical analyses will be conducted with the SAS^®^ System, version 9.3 or higher. The Intent to Treat (ITT) population will be the primary population for the efficacy analyses. Key secondary endpoints will be ranked a priori and tested sequentially with carry forward of the Type 1 error rate for rejected hypotheses. The change from baseline to the average of the 6 h and 9 h scores will be compared between treatment groups within an analysis of covariance model (ANCOVA) with the baseline SSS score, subject age group, the presence or absence of baseline neurotoxic symptoms, and completion of the initial dose of antivenom prior to the initiation of study drug as covariates.

Some patients meeting the criteria for inclusion in the trial may have completed the first dose of antivenom. By meeting inclusion criteria, these patients may still have significant sPLA2 toxicity and will be evaluable under the efficacy endpoints. The statistical plan includes analysis of efficacy endpoints with “completion of initial dose of antivenom prior to receipt of study drug (Yes or No)” as a covariate in the estimation models. Baseline SSS and the presence of neurotoxicity (SSS nervous system subscore 0–1 vs. 2–3) will also be included as covariates in the estimation models.

### 3.10. Ethics and Dissemination

This protocol was submitted to regulatory authorities in India and the US. A Notice to Proceed from the US Food and Drug Administration and Clinical Trial No Objection Certificate from the India Central Drugs Standard Control Organisation, Drug Controller General-India, have been obtained. At each site, a properly constituted IRB or EC reviewed and approved the protocol, the Investigator’s informed consent form (ICF), and related subject information and recruitment materials. This study will be conducted in compliance with the April 1996 ICH Guidance for Industry GCP E6, the Integrated Addendum to ICH E6 (R2) of November 2016, and the applicable regulations of the country in which the study is conducted. The trial was registered on Clinical trials.gov, NCT#04996264 and Clinical Trials Registry-India, 2021/07/045079 000062. Prior to performance of any study-related procedures, all patients, or legally authorized representative, must provide written consent by signing the ICF after being presented trial information, including procedures, other therapies available, rights, HIPAA compliance, and risks and benefits. Parents of pediatric patients must also consent to Parental Assent.

Results will be disseminated through peer-reviewed publications and conference presentations.

### 3.11. Patient and Public Involvement

This protocol was collaboratively designed to encompass treatment guidelines, practice conditions and resources that differ internationally. Contributions were made by clinical experts who have conducted patient-focused research related to snakebite envenoming resulting in key patient-reported outcomes being included as secondary outcomes.

### 3.12. Sample Size Calculation

A difference of 1 point in the snakebite severity score has previously been identified as the minimum clinically important difference [42]. The sample size of 94 patients was calculated using a formal calculation of 85% power, a two-sided alpha level of 5%, and a minimum expected difference between groups in the primary endpoint of 1.1 and standard deviation of 1.75. Allowing for 15 percent loss to follow-up, we plan to enroll up to 110 patients.

## 4. Discussion

The classification of snakebite envenoming as a Neglected Tropical Disease (NTD) by the World Health Organization (WHO) has underscored the urgency of the global health crisis posed by SBE [3]. WHO has established a multifaceted global strategy to improve the control, prevention, and treatment of SBE, including the development of new therapeutic approaches to treatment [14]. Because snake venom sPLA_2_ is involved in many venom effects and exists as a toxin of variable importance in >95% snake venoms [15]. sPLA_2_ has been identified as a desirable target for a toxin-specific approach to the treatment of snakebite [14,15].

Inhibition of snake venom sPLA_2_ by varespladib has the potential to overcome several key limitations of antivenom therapies. The extensive presence of sPLA_2_ in snake venoms supports the hypothesis that inhibition of sPLA_2_ could provide a broad-spectrum effect. In addition, antivenoms do not readily penetrate tissues or are often poorly matched to the venom antigens [2]. In contrast, varespladib is a DTI (molecular weight < 400 g/mol) with good tissue penetration due to its varied polar and hydrophobic structures [23].

To evaluate the clinical benefit of varespladib in the treatment of SBE, we developed the protocol for BRAVO (Broad-spectrum Rapid Antidote; Varespladib Oral for snakebite) as the first systematic clinical evaluation of a DTI for the treatment of SBE. The inclusion of pediatric patients ages 5 and older was supported by pre-clinical studies. If well-tolerated in this age group, future studies will consider including a younger population. Several items are included in this protocol that support the global nature of this study, including allowance for the 20WBCT within the hematologic SSS subscore for enrollment, enrollment of patients with SBE from any snake species, enrollment of those patients who have received antivenom, and modification of the SSS to include nephrotoxicity, thrombotic events, paralysis, and death.

The 20WBCT is frequently used outside of the U.S. as a rapid and easily performed assessment of coagulopathy to inform initial administration of antivenom. In the context of this clinical trial, the time to receive results from coagulation profiles would not reflect real world clinical practice and the delay would make many patients ineligible for enrollment. Others have considered how to effectively use the 20WBCT within controlled clinical studies given known false positive rates [43,44]. Here, we use 20WBCT to support inclusion in the trial, while efficacy will be evaluated with confirmatory laboratory tests. To minimize false positive rates, we have confirmed that all study sites in India perform the 20WBCT per Standard Treatment Guidelines [45]. Results for this study may help inform future clinical studies that also include 20WBCT analysis.

To our knowledge, BRAVO is the first international clinical trial to enroll patients bitten by a snake of any type rather than specific species or families. This protocol was collaboratively designed to accommodate differing snake species, treatment guidelines, and approaches. An important element of the study was to include as many severely ill patients as possible rather than focusing on selection of mild or moderately envenomed patients. This goal is achieved by allowing both trial arms (varespladib and placebo) to receive SoC concurrently. Thus, critically ill adult and pediatric patients may receive emergency treatment in a timely manner while being evaluated for the potential benefit associated with administration of a DTI such as varespladib-methyl.

The final major study design innovation is the use of a single, composite primary efficacy endpoint to characterize a range of global SBE syndromes that are sensitive to change over time and accepted by multiple regulatory authorities. As the first international clinical trial for SBE, the primary endpoint was designed with guidance from global knowledge leaders and regulatory authorities from both the US and India to evaluate clear clinical benefit based on an SSS intended for broader international use. The measurement of the primary endpoint at 6 and 9 h post study drug administration was selected to evaluate if varespladib confers benefit over SoC alone and was selected with advisement from regulatory agencies.

There is a clear need for improved compositive primary outcomes that can be used in clinical trials recruiting participants with diverse SBE syndromes. Application of an updated SSS by itself and integration with approaches such as the Core Outcomes Set in Snakebite [44] should be considered a priority [44]. These combined efforts have the potential to advance the field by facilitating more direct comparisons between clinical trial results independent of intervention or SBE syndrome. These novel trial design approaches such as use of the 20WBCT, enrollment of any venomous snake species, including those patients who have received antivenom, and use of a composite primary outcome that better reflects important clinical outcomes globally, will inform future SBE clinical trial design.

One potential limitation of our design is the challenge of using a composite primary endpoint that can measure the acute changes of clinically important outcomes such as neurotoxicity while accounting for clinically important differences in more gradually recovering systems such as those seen with venom-induced tissue injury. For example, the local wound subgroup of the SSS often takes days or weeks to improve. To explore this, we have added an area under the curve (AUC) through 7 days as a secondary endpoint to resolve improvements in systems that recover at different rates. Additionally, more improvements are needed to the SSS, although it is a useful basis for a composite endpoint in a snakebite study. In particular, specific scores are needed for pediatric subjects that account for smaller body size and normal vital signs in children and, as well, better means of discrimination between weakness and other neurological syndromes within the neurological subsection. Another potential limitation of the study is related to the general asymmetry of subscores within the SSS. For example, the neurotoxicity subscore is on a three-point scale while the local wound and hematologic system subscores are on a four-point scale. These limitations can be somewhat mitigated by isolated analyses of the subscores as secondary endpoints. Further, the neurological subscore measures are subjective and do not clearly discriminate between weakness and non-weakness syndromes and may need to be updated for future studies. Variations in ventilatory support management and investigator scoring under conditions that include sedation and its removal for clinical examination may obscure scoring. Future studies may further consider adding electrophysiological measures for such patients. The goal of the secondary endpoints in this protocol is to assess system-specific toxicities as well as extend the clinical findings over the full study period to mitigate limitations and augment conclusions from the primary endpoint. Because snakebite trials are so rare and challenging, these endpoints were selected to maximize amount of information obtained in evaluating a novel therapy whose clinical impact for SBE is still uncertain. Additionally, these secondary endpoints will allow for a more complete psychometric evaluation of our primary endpoint and may provide useful baselines for more longitudinal studies of snakebite envenoming victims [46,47,48,49,50,51,52,53,54].

If proven efficacious as an adjunct to SoC in a hospital setting, future studies may examine the efficacy of oral varespladib or other novel DTIs or antibody formats to be administered in the prehospital setting or outpatient clinic. Lack of proximity and/or access to healthcare and antivenom, and delays in receiving care severely impact outcomes [3,5,6,7]. The WHO’s Snakebite Envenoming Working Group stated that “accelerating preclinical and clinical testing of promising prehospital adjunctive treatments, such as the phospholipase A2 inhibitor varespladib or varespladib-methyl, as part of the WHO snake bite envenoming research agenda may lead to early improvements in prehospital survival” [14]. Early administration of DTIs at the time of the snakebite may be sufficient to delay many severe effects of SBE, thereby augmenting or replacing existing antivenom therapy.

In conclusion, if successful, the BRAVO trial design may begin to help develop templates for future trials of novel treatments for SBE that allows for direct comparison between clinical trial results independent of intervention or SBE syndrome, some structures for which others have recently focused, and are, as well beginning clinical studies such as with unithiol [44,55]. This trial’s design and future iterations should allow for the clinical evaluation of oral varespladib and other candidate therapies with SoC in patients with SBE from diverse snake species found in India and the US. Results from this trial hold the potential for developing broad-spectrum SBE treatments that overcomes several key limitations of current therapies with the goal of making significant contributions toward meeting the WHO goal of reducing death and disability by half by 2030 [14].

## Figures and Tables

**Table 1:** Patient Eligibility.

Inclusion Criteria					
Male or female					
≥5 years of age					
Presence of a symptomatic venomous snakebite					
Symptom onset within 10 h of eligibility assessment					
Willingness to provide informed consent prior to initiation of study procedure					
Patients must meet inclusion criteria in one of two categories:					
Category 1: Patient has not completed first dose of antivenom			Category 2: Patient has completed initial dose of antivenom		
SSS inclusion score			SSS inclusion score		
≥2 in one system and ≥1 in another system			≥2 in one system and ≥1 in another system		
OR			OR		
≥3 in at least one system			≥3 in at least one system		
			AND		
			CGI-I score ≥ 5		
* Local wound, pulmonary, cardiovascular, hematologic, or nervous system scores only. GI and renal scores are not used as inclusion criteria.					
Exclusion Criteria					
Clinically significant upper GI bleed History of cerebral vascular accident or intracranial bleeding of any kind History of acute coronary syndrome Severe pulmonary hypertension			Known history of inherited bleeding or coagulation disorder, Chronic liver disease Chronic kidney disease On anticoagulation therapy Pregnant and/or breast-feeding Known allergy to varespladib		

**Table 2:** Modified Snakebite Severity Score Intended for International Use.

Local Wound #	
No signs/symptoms	0
Pain, swelling, or ecchymosis within 5–7.5 cm of bite site	1
Pain, swelling, or ecchymosis involving less than half the extremity (7.5–50 cm from bite site)	2
Pain, swelling, or ecchymosis involving half to all of extremity (50–100 cm from bite site)	3
Pain, swelling, or ecchymosis extending beyond affected extremity (more than 100 cm of bite site)	4
Pulmonary symptoms #,*	
No signs/symptoms	0
Dyspnea, minimal chest tightness, mild/vague discomfort, respirations of 20–25 breaths per minute	1
Moderate respiratory distress, 26–40 bpm	2
Cyanosis, air hunger, extreme tachypnea, or respiratory insufficiency/failure	3
Cardiovascular system #,*	
No signs/symptoms	0
HR 100–125 BPM, palpitations, generalized weakness, benign dysrhythmia, or hypertension	1
HR 126–175 BPM, or hypotension with SBP > 100 mmHg	2
HR > 175 BPM, or hypotension with SBP < l00 mmHg, malignant dysrhythmia, or cardiac arrest	3
**Gastrointestinal system**	
No signs/symptoms	0
Pain, tenesmus, or nausea	1
Vomiting or diarrhea	2
Repeated vomiting, diarrhea, hematemesis, or hematochezia	3
Hematologic symptoms #,*	
No signs/symptoms	0
Coagulation parameters slightly abnormal: PT ULN–20 s, PTT ULN–50 s, platelets 100–150 K/mL, or fibrinogen 100–150 mcg/mL	1
Coagulation parameters abnormal: PT 20–50 s, PTT 50–75 s, platelets 50–100 K/mL, or fibrinogen 50–100 mcg/mL	2
Coagulation parameters abnormal: PT 50–100 s, PTT 75–100 s, platelets 20–50 K/mL, or fibrinogen <50 mcg/mL	3
Coagulation parameters markedly abnormal, with serious bleeding or the threat of spontaneous bleeding; unmeasurable PT or PTT, platelets <20 K/mL, undetectable fibrinogen, severe abnormalities of other laboratory values also fall into this category	4
Nervous system #,*	
No signs/symptoms	0
Minimal apprehension, headache, weakness, dizziness, chills, or paresthesia	1
Moderate apprehension, headache, weakness, dizziness, chills, paresthesia, confusion, fasciculation in area of bite site, ptosis, or dysphagia	2
Severe confusion, lethargy, weakness, paralysis, seizures, coma, psychosis, or generalized fasciculation	3
Renal system *	
Normal creatinine and urine output	0
Creatinine 1.5 to 1.9 times baseline, increase in creatinine ≥0.3 mg/dL (≥26.5 µmol/L) from baseline, or urine output <0.5 mL/kg/h for >6 h	1
Creatinine 2 to 2.9 times baseline or urine output <0.5 mL/kg/h for >12 h	2
Creatinine ≥3.0 times baseline, increase in creatinine to ≥4.0 mg/dL (≥353.6 µmol/L), urine output <0.3 mL/kg/h for ≥24 h or anuria ≥12 h, or initiation of renal replacement therapy	3
**TOTAL**	

# Subscores used for inclusion determination. Hematologic subscore may be used if available, but enrollment should not be delayed. Abnormal 20WBCT may be used for enrolment determinations for the Hematologic system, if performed per site standard of care. Severe abnormalities of other coagulation parameter laboratory values, including 20WBCT, should be scored as a 4 in the Hematologic system subscore for purposes of enrolment. * Subscores used for primary endpoint. Only definitive laboratory testing will be used for efficacy endpoints.

**Table 3:** Abbreviated Schedule of Assessments.

Assessment	Day 1					Day 2	Day 3	Day 7	Day 14	Day 28
	Baseline	0–2 h	3–4 h	5–6 h	8–10 h			±1 day	±2 days	EOS
Individual SSS category severity ^a^										
Head-lift duration (0–5 s) ^b^										
NPRS										
Grip strength										
Laboratory assessments										
Liver Function Tests										
CGI-I ^c^										
PGIC										
PSFS										
C-SSR										
12-lead ECG										

Colored boxes indicate scheduled visit assessment. ^a^ Pulmonary, cardiovascular, local wound, gastrointestinal, hematologic, renal, and nervous system sections. SSS measurements at each day 1 timepoint to be collected prior to administration of repeat doses of antivenom. ^b^ Inpatients. ^c^ Day 1 assessments performed at 1, 2 and 3–4 h after initial dose of varespladib or placebo. Abbreviations; CGI-I = clinical global impressions scale-improvement subscale; C-SSR = Columbia suicide severity rating scale; ECG = electrocardiogram; NPRS = Numeric pain rating scale; PGIC = Patient global impression of change; PSFS = patient-specific functional scale; SSS = snakebite severity score.

**Table 4:** Primary and Secondary Efficacy Outcomes.

Primary Objective: To Evaluate the Efficacy of a Multidose Regimen of Oral Varespladib-Methyl with SoC in Subjects after Venomous Snakebites
Change in the composite outcome of pulmonary, cardiovascular, hematologic symptoms, renal, and nervous system sections of the modified SSS from baseline to the average of the score from 6 and 9 h after first dose
**Secondary Objectives: To evaluate efficacy of varespladib-methyl** **as treatment for sPLA_2_-induced venom toxicities**
AUC of the pulmonary, cardiovascular, hematologic symptoms, renal, and nervous system sections of the modified SSS from baseline through day 7
Complete SSS from baseline through day 7
SSS neurologic system subscore from baseline through day 3
Coagulation abnormalities from baseline through day 3
Hemolysis markers from baseline through day 3
Levels of the myonecrosis marker, creatine kinase from baseline through day 3
Numeric Pain Rating score in patients able to respond from baseline through day 28
Kidney function markers from baseline through day 28
Total differential antivenom requirement from baseline through day 28
Head-lift duration from baseline through day 7
Total duration of ventilatory support from baseline through day 28
Total duration of Intensive Care Unit stay from baseline through day 28
Total duration of hospitalization from baseline through day 28
All-cause mortality from baseline through day 28
Clinical Global Impression-Improvement from baseline through day 7
Patient Global Impression of Change from baseline through day 7
Patient-specific Functional Scale total score from baseline through day 28

**Table 5:** Safety Endpoints.

Incidence and severity of adverse events (AEs), serious AEs (SAEs), and AEs leading to discontinuation of Investigational Product (IP)
Safety of varespladib-methyl as assessed by the number and rates of reported treatment-emergent adverse events (TEAEs) from beginning of treatment until last Follow-Up Visit/Telephone Call at Day 28
Number of subjects with a treatment-related SAE from beginning of treatment until last Follow-Up Visit/Telephone Call at Day 28
Safety as assessed by Vital signs Clinical laboratory evaluations: CBC, urinalysis, liver function tests [LFTs], renal function tests (albumin, creatinine, blood urea nitrogen [BUN], estimated glomerular filtration rate [eGFR]) 12-lead electrocardiogram (ECG)
Concomitant medications and therapies
Columbia-Suicide Severity Rating Scale (C-SSRS) * evaluated at Baseline or at the earliest time point clinically allowable (ideally Day 1) and then at every study visit through Day 28

* The Columbia-Suicide Severity Rating Scale is typically requested for all investigational drugs reviewed by the US Food and Drug Administration Division of Neurology.

## Data Availability

BRAVO study data will be held by Ophirex, Inc. and data from the study that is published will be published in open access journals for transparency and accessibility.

## Supplementary Materials

The following supporting information can be downloaded at: https://www.mdpi.com/article/10.3390/toxins15010022/s1, Table S1: IRB and Ethics Committee Approvals; Table S2: Schedule of Events; Table S3: Original Snakebite Severity Score; Table S4: Reporting checklist for protocol of a clinical trial; File S1: BRAVO Study Group.
