# Supplementary material for: The BRAVO Clinical Study Protocol: Oral Varespladib for Inhibition of Secretory Phospholipase A2 in the Treatment of Snakebite Envenoming
Source: Toxins (Basel). 2022 Dec 28;15(1):22. doi: 10.3390/toxins15010022 (PMC9862656; doi:10.3390/toxins15010022)
Supplement: Supplementary file 1 [file toxins-15-00022-s001.zip › toxins-2085267-supplementary.pdf]

# Supplementary Materials: Protocol for the BRAVO Multinational Randomized Trial: Oral Varespladib a Secretory Phospholipase $A_2$ Inhibitor, for the Treatment of Snakebite Envenoming

**Rebecca W. Carter, Charles J. Gerardo, Stephen P. Samuel, Surendra Kumar, Suneetha D.K., Partha P. Mukherjee, Farshad M. Shirazi, Peter D. Akpunonu, Chanaveerappa Bammigatti, Ashish Bhalla, Neeraj Manikath, Timothy F. Platts-Mills and Matthew R. Lewin on behalf of the BRAVO study group**

**Supplementary Table S1.** IRB and Ethics Committee Approvals.

Ethics committees/IRBs of the following listed institutions gave ethical approval for this work

*US IRB Approvals*

| Site # | IRB Name | IRB Reference # |
|---|---|---|
| SB1-001 | Duke University Health System IRB | PRO00108196-INT-1.0 |
| SB1-002 | Medical IRB Office of Research Integrity; University of Kentucky | 67486 |
| SB1-003 | Institutional Review Board; Loma Linda University Medical Center | 5210243 |
| SB1-004 | University of Arizona Human Subjects Protection Program | MOD00000199 |
| SB1-005 | University of Mississippi Medical Center IRB | MOD00004967_STUDY00001829 |
| SB1-006 | WCG IRB; University of Florida Health - Jacksonville | 1319858 |
| SB1-007 | WCG IRB; Augusta University Medical Center | 1317849 |
| SB1-009 | Louisiana State University Health Sciences Center Shreveport IRB | STUDY00001829 |

*India Ethics Committee Approvals*

| Site No. | IEC name and Address | DCGI Registration No. | Study Reference No. |
|---|---|---|---|
| SB1-011 | Ethics committee, S.P. Medical college<br>Bikaner-334003 | ECR/27/SP/Inst/RJ/2013/RR-19 | OPX-PR-01 |
| SB1-012 | Postgraduate institute of medical education and research, Chandigarh<br>Institutional ethics committee | ECR/25/Inst/CH/2013/RR-20 | CT-09/2021-116 |
| SB1-013 | Ethics committee, Calcutta national medical college (EC-CNMC)<br>32, Gorachand road, Kolkata - 14, West Bengal, India | ECR/771/Inst/WB/2015/RR-18 | EC-CNMC/ 2022/2-A |
| SB1-014 | Institutional ethics committee -interventional studies<br>JIPMER, Puducherry | ECR/342/Inst/PY/2013/RR-19 | JIP/IEC/2021/081 |
| SB1-016 | Institutional ethics committee<br>Government medical college Kozhikode | ECR/395/Inst/KL/2013/RR-20 | OPX/ecapr04/07/2022 |
| SB1-018 | Institutional ethics committee, Mysore medical college and research institute<br>And associated hospitals, Mysuru - 570 001 | ECR/134/lnst/KA/2013/RR-19 | MMC EC 45/22 |
| SB1-019 | Father muller institutional ethics committee (fmiec),<br>Father muller road, Kankanady, Mangalore - 575 002, Karnataka, India | ECR/540/Inst/KA/2014/RR-20 | 41O/2O22 |
| SB1-021 | Institutional ethics committee (IEC)<br>Department of pharmacology,<br>Govt. Medical college, Nagpur | ECR/43/Inst/MH/2013/RR-22 | 3768/EC/Pharmac/GMC/NGP/ |

**Supplementary Table S2.** Schedule of Events.

| **Visit**[a] | **Baseline (Day 1)** | **Dosing (Day 1)** | **Day 1**[c] | | | | | **Day 2**[d] | **Day 3** | **Day 7** | **Day 14** | **Day 28 EOS Visit** |
|---|---|---|---|---|---|---|---|---|---|---|---|---|
| **Time After Dosing**[e] | **Pre-dosing** | **0h** | **0.5–1h** | **3–4h** | **5–6h** | **8–10h** | **12h** | **2d** | **3d** | **7d ± 1d** | **14d ± 2d** | **28d ± 3d** |
| Informed consent | X | | | | | | | | | | | |
| Review inclusion / exclusion criteria | X | | | | | | | | | | | |
| Demographics | X[b] | | | | | | | | | | | |
| Medical history, including details of envenoming and treatments/interventions | X | | | | | | | | | | | |
| Body height/weight per institutional protocol | X[b] | | | | | | | | | | | X |
| Physical examination | X | | | | | | | X | X | X | X | X |
| Vital signs[f] | X | | | X | X | X | | X | X | X | X | X |
| Randomization and IP assignment | X | | | | | | | | | | | |
| Snakebite Severity Scale (SSS) assessments:<br><br>Individual Category Severity Assessments: Pulmonary, Cardiovascular, Local Wound, Gastrointestinal, Hematologic, Renal, Nervous Systems | X | | | X[g] | X[g] | X[g] | | X | X | X | X | X |
| Head-Lift duration in inpatient participants (0 to 5 seconds)[h] | X | | | X | X | X | | X | X | X | X | X |
| Numeric Pain Rating Scale (NPRS) | X | | | X | X | X | | X | X | X | X | X |
| 12-lead ECG | X[b] | | | | | X | | | | X | | X |
| Grip strength[i] | X | | | X | X | X | | X | X | X | X | X |
| Laboratory assessments (see individual Laboratory Assessments Schedule of Events below)[j] | X | | | X | X | X | | X | X | X | X | X |
| Patient Global Impression of Change (PGIC)[k] | X[b] | | | X | X | X | | X | X | X | X | X |
| Clinical Global Impression-Improvement (CGI-I)[l] | X[l] | | | | X | X | | X | X | X | X | X |
| Patient-specific functional scale (PSFS) | | | | | | X | | X | X | X | X | X |
| Columbia-Suicide Severity Rating Scale (C-SSRS)[m] | X[b] | | | | | | | X | X | X | X | X |

| Visit[a] | Baseline (Day 1) | Dosing (Day 1) | Day 1[c] | | | | | Day 2[d] | Day 3 | Day 7 | Day 14 | Day 28 EOS Visit |
|---|---|---|---|---|---|---|---|---|---|---|---|---|
| **Time After Dosing[e]** | **Pre-dosing** | **0h** | **0.5–1h** | **3–4h** | **5–6h** | **8–10h** | **12h** | **2d** | **3d** | **7d ± 1d** | **14d ± 2d** | **28d ± 3d** |
| IP administration (Oral loading dose followed by second dose approximately 12 hours later, then BID dosing for remainder of 7-day treatment period) | | ← | | | | | | | → | | | |
| Standard of care (SOC)[n] | ← | | | | | | | | | | | → |
| Assess and record adverse events (AEs) | ← | | | | | | | | | | | → |
| Record concomitant medications/therapies/analgesic use, including sedatives and paralytics for intubated patients | ← | | | | | | | | | | → | |
| Additional assessments in intubated subjects[o] | ← | | | | | | | | → | | | |
| Record pulmonary support interventions[p] | ← | | | | | | | | | | → | |
| Record hospitalization details | ← | | | | | | | | | | | → |
| **Laboratory Assessments Schedule of Events** | | | | | | | | | | | | |
| Complete blood count (including WBCs with differential, hemoglobin/hematocrit, platelets) | X | | | X | X | X | | X | X | X | X | X |
| Serum chemistry (including sodium, potassium, chloride, bicarbonate, BUN and creatinine with estimated GFR) | X | | | X | X | X | | X | X | X | X | X |
| Serum pregnancy | X | | | | | | | | | | | X |
| Liver function tests (LFTs) | X | | | | X | | | X | X | X | | X |
| Hemolysis markers (hemolysis measured by plasma free hemoglobin, haptoglobin, LDH) | X | | | X | X | X | | X | X | | | |
| PT, PTT, thrombin time, INR[q] | X | | | X | X | X | | X | X | X | X | X |
| Absolute fibrinogen | X | | | X | X | X | | X | X | X | X | X |
| Urinalysis, general[r] | X | | | | | | | X | X | X | | X |
| Urine pregnancy | X | | | | | | | | | | | |
| Biomarkers: CRP, D-dimer | X | | | | | X | | X | X | X | X | |
| Biomarker: creatine kinase (CK) | X | | | X | X | X | | X | X | | | |
| $sPLA_2$ biomarker[s] | X | | X | X | X | X | | X | X | X | | |
| Pharmacokinetics samples[s,t] | X | | X[s] | | | | | | X | X | | |

Abbreviations: AE = adverse event; BID = twice daily; BUN = blood urea nitrogen; CGI-I = Clinical Global Impression-Improvement; CK = creatine kinase; CRP = C-reactive protein; C-SSRS = Columbia-Suicide Severity Rating Scale; ECG = electrocardiogram; EOS = End of Study; GFR = glomerular filtration rate; INR = international normalized ratio; IP = investigational product; LDH = lactate dehydrogenase; MIP = maximum inspiratory pressure; NIF = negative inspiratory force; NPRS = numeric pain rating scale; PGIC = patient global impression of change; PK = pharmacokinetic; PSFS = patient-specific functional scale; PT = prothrombin time; PTT = partial thromboplastin time; SOC = Standard of care; sPLA2 = secretory phospholipase A2; SSS = snakebite severity score; WBC = white blood cell

[a] Screening, enrollment, and Day 1 of the Treatment Period will take place on the same day.

[b] This noncritical assessment should be performed as early as possible, but may be performed after randomization and initiation of study drug or SOC if required due to patient condition

[c] Assessments (besides PK blood draws for each individual subject on Day 1) should be timed such that ≥ 90 minutes elapses between measurements.

[d] Assessments for each individual subject between hours 8 to 10 of Day 1 and the same measurements on Day 2 (except PK blood draws) should be timed such that at least 12 and not more than 18 hours elapse between measurements.

[e] Assessments for each individual subject between Day 2 and Day 3 should be timed such that approximately, but not more than, 24 hours elapse between measurements

[f] Vital signs will be assessed per institutional practice, but at a minimum of once per time point.

[g] The SSS measurements at each of the Day 1 timepoints (3 to 4 hours, 5 to 6 hours, and 8 to 10 hours post-dose) should be collected prior to administration of repeat doses of antivenom, if required.

[h] Head-lift will be evaluated from 0 to 5 seconds .

[i] Grip strength should be performed using a dynamometer, as a measure of the severity of the neurological effects of snakebite .

[j] Administration of treatment should **not** be delayed until laboratory test results are returned (e.g., eGFR, liver, renal, or serum pregnancy tests).

[k] The PGIC score is a rapid, qualitative assessment that should be performed prior to the PSFS score on Day 1

[l] Baseline CGI-I should be in comparison to previous clinical assessment. Additional CGI-I should be performed on Day 1 at 1, 2, and 3-4 hours post initial dose, then follow normal assessment schedule. All post-baseline CGI-I assessments should be compared to baseline CGI-I.

[m] The C-SSRS will be evaluated at Baseline or at the earliest time point clinically allowable and then at every study visit through Day 28, with modifications to the scales at different study dates.

[n] SOC (including antivenom as required) will continue to be administered throughout the subject's participation in the study according to the protocol and the judgment of the Investigator.

[o] For endotracheally intubated subjects, remove sedation (and if applicable, paralytics) sufficiently early for awake examination (e.g. until patient is sufficiently awake to follow commands) per institutional practice, then record SSS and Head-Lift duration. In subjects who are unable to achieve an acceptable level of alertness 60 minutes after discontinuation of sedation (and if applicable, paralytics), record SSS and Head-Lift duration. .

[p] Record the type of control of mechanical ventilation [e.g., volume control, pressure control, assist control, pressure-regulated volume control, synchronized intermittent mandatory ventilation, pressure support, adaptive support ventilation, airway pressure release ventilation or T piece, note NIF (MIP) values in eCRF as used per institutional practice].

[q] Results of point of care testing (e.g., 20WBCT) will also be collected when performed per site standard of care.

[r] Urinalysis should not delay care and should not be obtained by catheter unless patient already being catheterized (e.g., Russell's viper). Urine from catheterized patients should not be collected from bag, but rather proximal port.

[s] $sPLA_2$ sampling will be performed for all subjects on Day 1 (pre-dose and 1, 3, 6, and 9 hours post-first dose), and on Day 2, Day 3, and Day 7 (pre-dose). $sPLA_2$ in serum and PK samples will be processed and analyzed in a central laboratory, not at individual sites.

[t] PK sampling will only be performed for a total of 20 adult and pediatric subjects on Days 1 and 3, and all subjects on Day 7. Once 20 subjects have provided PK samples for Days 1 and 3, sites will be notified by the sponsor to discontinue Day 1 and 3 PK sampling. Subjects will be enrolled for PK analysis as follows: Day 1 - 20 subjects, pre-dose and 0.5, 1, 2, 4, 6, 8, and 12 (pre-second dose) hours; Day 3 - 20 subjects, pre-dose and 0.5, 1, 2, 4, 6 hours; Day 7 - all subjects, pre-dose.

**Supplementary Table S3.** Original Snakebite Severity Score (published with author's permission, Dart, R.C.; Hurlbut, K.M.; Garcia, R.; Boren, J. Validation of a Severity Score for the Assessment of Crotalid Snakebite. Ann. Emerg. Med. 1996, 27, 321–326. [CrossRef] [PubMed])).

| | |
|---|---|
| **Local wound #** | |
| No symptoms/signs | 0 |
| Pain, swelling, or ecchymosis within 5–7.5 cm of bite site | 1 |
| Pain, swelling, or ecchymosis involving less than half the extremity (7.5–50 cm from bite site) | 2 |
| Pain, swelling, or ecchymosis involving half to all of extremity (50–100 cm from bite site) | 3 |
| Pain, swelling, or ecchymosis extending beyond affected extremity (more than 100 cm of bite site) | 4 |
| **Pulmonary symptoms** | |
| No symptoms/signs | 0 |
| Dyspnea, minimal chest tightness, mild/vague discomfort, respirations of 20–25 | 1 |
| Moderate respiratory distress (tachypnea, 26 to 40 breaths/minute; accessory muscle use) | 2 |
| Cyanosis, air hunger, extreme tachypnea, or respiratory insufficiency /failure | 3 |
| **Cardiovascular system** | |
| No symptoms/signs | 0 |
| Tachycardia 1100 to 125 beats/minute), palpitations, generalized weakness, benign dysrhythmia, or hypertension | 1 |
| Tachycardia 1126 to 175 beats/minute) or hypotension, with systolic blood pressure greater than 100 mm Hg | 2 |
| Extreme tachycardia I> 175 beats/minute), hypotension with systolic blood pressure <100 mm Hg, malignant dysrhythmia, or cardiac arrest | 3 |
| **Gastrointestinal system** | |
| No symptoms/signs | 0 |
| Pain, tenesmus, or nausea | 1 |
| Vomiting or diarrhea | 2 |
| Repeated vomiting, diarrhea, hematemesis, or hematochezia | 3 |
| **Hematologic symptoms** | |
| No symptoms/signs | 0 |
| Coagulation parameters slightly abnormal: PT, <20 seconds; PTT, <50 seconds; platelets, 100,000 to 150,000/ml; or fibrinogen, 100 to 150 µg/ml | 1 |
| Coagulation parameters abnormal: PT, <20 to 50 seconds; PTT, <50 to 75 seconds; platelets, 50,000 to 100,000/ml; or fibrinogen, 50 to 100 µg/ml | 2 |
| Coagulation parameters abnormal: PT, <50 to 100 seconds; PTT, <75 to 100 seconds; platelets, 20,000 to 50,000/ml; or fibrinogen, <50 µg/ml | 3 |
| Coagulation parameters markedly abnormal, with serious bleeding or the threat of spontaneous bleeding: unmeasurable PT or PTT; platelets, <20,000/ml; or undetectable fibrinogen; severe abnormalities of other laboratory values also fall into this category | 4 |
| **Central nervous system #,*** | |
| No symptoms/signs | 0 |
| Minimal apprehension, headache, weakness, dizziness, chills, or paresthesia | 1 |
| Moderate apprehension, headache, weakness, dizziness, chills, paresthesia, confusion, or fasciculation in area of bite site | 2 |
| Severe confusion, lethargy, seizures, coma, psychosis, or generalized fasciculation | 3 |

**Supplementary Table S4. Standard Protocol Items: Recommendations for Interventional Trials (SPIRIT)** Reporting checklist for protocol of a clinical trial.

| Administrative information | | SPIRIT Reporting Item | Page Number |
|---|---|---|---|
| Title | #1 | Descriptive title identifying the study design, population, interventions, and, if applicable, trial acronym | 1 |
| Trial registration | #2a | Trial identifier and registry name. If not yet registered, name of intended registry | 1, 12 |
| Trial registration: data set | #2b | All items from the World Health Organization Trial Registration Data Set | 2-12 |
| Protocol version | #3 | Date and version identifier | Provided in registry |
| Funding | #4 | Sources and types of financial, material, and other support | 14 |
| Roles and responsibilities: contributorship | #5a | Names, affiliations, and roles of protocol contributors | 1, Appendix |
| Roles and responsibilities: sponsor contact information | #5b | Name and contact information for the trial sponsor | 1 |
| Roles and responsibilities: sponsor and funder | #5c | Role of study sponsor and funders, if any, in study design; collection, management, analysis, and interpretation of data; writing of the report; and the decision to submit the report for publication, including whether they will have ultimate authority over any of these activities | 1, 14 |
| Roles and responsibilities: committees | #5d | Composition, roles, and responsibilities of the coordinating centre, steering committee, endpoint adjudication committee, data management team, and other individuals or groups overseeing the trial, if applicable (see Item 21a for data monitoring committee) | 10 |
| **Introduction** | | | |
| Background and rationale | #6a | Description of research question and justification for undertaking the trial, including summary of relevant studies (published and unpublished) examining benefits and harms for each intervention | 1-4 |
| Background and rationale: choice of comparators | #6b | Explanation for choice of comparators | 7-8 |
| Objectives | #7 | Specific objectives or hypotheses | 4 |
| Trial design | #8 | Description of trial design including type of trial (eg, parallel group, crossover, factorial, single group), allocation ratio, and framework (eg, superiority, equivalence, non-inferiority, exploratory) | 4 |
| **Methods: Participants, interventions, and outcomes** | | | |
| Study setting | #9 | Description of study settings (eg, community clinic, academic hospital) and list of countries where data will be collected. Reference to where list of study sites can be obtained | 4 |
| Eligibility criteria | #10 | Inclusion and exclusion criteria for participants. If applicable, eligibility criteria for study centres and | 4, Table 1 |

| | | | |
|---|---|---|---|
| | | individuals who will perform the interventions (eg, surgeons, psychotherapists) | |
| Interventions: description | #11a | Interventions for each group with sufficient detail to allow replication, including how and when they will be administered | 7 |
| Interventions: modifications | #11b | Criteria for discontinuing or modifying allocated interventions for a given trial participant (eg, drug dose change in response to harms, participant request, or improving / worsening disease) | 7 |
| Interventions: adherance | #11c | Strategies to improve adherence to intervention protocols, and any procedures for monitoring adherence (eg, drug tablet return; laboratory tests) | N/A, primarily emergency department protocol |
| Interventions: concomitant care | #11d | Relevant concomitant care and interventions that are permitted or prohibited during the trial | 4 |
| Outcomes | #12 | Primary, secondary, and other outcomes, including the specific measurement variable (eg, systolic blood pressure), analysis metric (eg, change from baseline, final value, time to event), method of aggregation (eg, median, proportion), and time point for each outcome. Explanation of the clinical relevance of chosen efficacy and harm outcomes is strongly recommended | 8, Table 4 |
| Participant timeline | #13 | Time schedule of enrolment, interventions (including any run-ins and washouts), assessments, and visits for participants. A schematic diagram is highly recommended (see Figure) | Table 3 |
| Sample size | #14 | Estimated number of participants needed to achieve study objectives and how it was determined, including clinical and statistical assumptions supporting any sample size calculations | 12 |
| Recruitment | #15 | Strategies for achieving adequate participant enrolment to reach target sample size | N/A, emergency department protocol |
| **Methods: Assignment of interventions (for controlled trials)** | | | |
| Allocation: sequence generation | #16a | Method of generating the allocation sequence (eg, computer-generated random numbers), and list of any factors for stratification. To reduce predictability of a random sequence, details of any planned restriction (eg, blocking) should be provided in a separate document that is unavailable to those who enrol participants or assign interventions | 7 |
| Allocation concealment mechanism | #16b | Mechanism of implementing the allocation sequence (eg, central telephone; sequentially numbered, opaque, sealed envelopes), describing any steps to conceal the sequence until interventions are assigned | 7 |
| Allocation: implementation | #16c | Who will generate the allocation sequence, who will enrol participants, and who will assign participants to interventions | 7 |

| | | | |
|---|---|---|---|
| Blinding (masking) | #17a | Who will be blinded after assignment to interventions (eg, trial participants, care providers, outcome assessors, data analysts), and how | 7 |
| Blinding (masking): emergency unblinding | #17b | If blinded, circumstances under which unblinding is permissible, and procedure for revealing a participant's allocated intervention during the trial | 7 |
| **Methods: Data collection, management, and analysis** | | | |
| Data collection plan | #18a | Plans for assessment and collection of outcome, baseline, and other trial data, including any related processes to promote data quality (eg, duplicate measurements, training of assessors) and a description of study instruments (eg, questionnaires, laboratory tests) along with their reliability and validity, if known. Reference to where data collection forms can be found, if not in the protocol | 7-8 |
| Data collection plan: retention | #18b | Plans to promote participant retention and complete follow-up, including list of any outcome data to be collected for participants who discontinue or deviate from intervention protocols | N/A – emergency department protocol |
| Data management | #19 | Plans for data entry, coding, security, and storage, including any related processes to promote data quality (eg, double data entry; range checks for data values). Reference to where details of data management procedures can be found, if not in the protocol | 11 |
| Statistics: outcomes | #20a | Statistical methods for analysing primary and secondary outcomes. Reference to where other details of the statistical analysis plan can be found, if not in the protocol | 11 |
| Statistics: additional analyses | #20b | Methods for any additional analyses (eg, subgroup and adjusted analyses) | 11 |
| Statistics: analysis population and missing data | #20c | Definition of analysis population relating to protocol non-adherence (eg, as randomised analysis), and any statistical methods to handle missing data (eg, multiple imputation) | 11 |
| **Methods: Monitoring** | | | |
| Data monitoring: formal committee | #21a | Composition of data monitoring committee (DMC); summary of its role and reporting structure; statement of whether it is independent from the sponsor and competing interests; and reference to where further details about its charter can be found, if not in the protocol. Alternatively, an explanation of why a DMC is not needed | 8 |
| Data monitoring: interim analysis | #21b | Description of any interim analyses and stopping guidelines, including who will have access to these interim results and make the final decision to terminate the trial | N/A – no interim analysis included |
| Harms | #22 | Plans for collecting, assessing, reporting, and managing solicited and spontaneously reported adverse events and other unintended effects of trial interventions or trial conduct | 8, Table 4c |

| | | | |
|---|---|---|---|
| Auditing | #23 | Frequency and procedures for auditing trial conduct, if any, and whether the process will be independent from investigators and the sponsor | Not in paper, 11 -12 (GCP and regulatory compliance) |
| **Ethics and dissemination** | | | |
| Research ethics approval | #24 | Plans for seeking research ethics committee / institutional review board (REC / IRB) approval | 11-12 |
| Protocol amendments | #25 | Plans for communicating important protocol modifications (eg, changes to eligibility criteria, outcomes, analyses) to relevant parties (eg, investigators, REC / IRBs, trial participants, trial registries, journals, regulators) | 11-12, Modifications through same process |
| Consent or assent | #26a | Who will obtain informed consent or assent from potential trial participants or authorised surrogates, and how (see Item 32) | 12 |
| Consent or assent: ancillary studies | #26b | Additional consent provisions for collection and use of participant data and biological specimens in ancillary studies, if applicable | 12 |
| Confidentiality | #27 | How personal information about potential and enrolled participants will be collected, shared, and maintained in order to protect confidentiality before, during, and after the trial | 11-12 |
| Declaration of interests | #28 | Financial and other competing interests for principal investigators for the overall trial and each study site | 15 |
| Data access | #29 | Statement of who will have access to the final trial dataset, and disclosure of contractual agreements that limit such access for investigators | 12 |
| Ancillary and post trial care | #30 | Provisions, if any, for ancillary and post-trial care, and for compensation to those who suffer harm from trial participation | 12 |
| Dissemination policy: trial results | #31a | Plans for investigators and sponsor to communicate trial results to participants, healthcare professionals, the public, and other relevant groups (eg, via publication, reporting in results databases, or other data sharing arrangements), including any publication restrictions | 12 |
| Dissemination policy: authorship | #31b | Authorship eligibility guidelines and any intended use of professional writers | 14 |
| Dissemination policy: reproducible research | #31c | Plans, if any, for granting public access to the full protocol, participant-level dataset, and statistical code | N/A |
| **Appendices** | | | |
| Informed consent materials | #32 | Model consent form and other related documentation given to participants and authorised surrogates | N/A |
| Biological specimens | #33 | Plans for collection, laboratory evaluation, and storage of biological specimens for genetic or molecular analysis in the current trial and for future use in ancillary studies, if applicable | N/A |

None The SPIRIT Explanation and Elaboration paper is distributed under the terms of the Creative Commons Attribution License CC-BY-NC. This checklist can be completed online using https://www.goodreports.org/, a tool made by the EQUATOR Network in collaboration with Penelope.ai

**Supplementary File S1.** BRAVO Study Group.

Michael Abouyannis
Centre for Snakebite Research and Interventions. Liverpool School of Tropical Medicine, Liverpool, UK
KEMRI-Wellcome Research Programme, Kilifi, Kenya

Richard C Dart
Rocky Mountain Poison and Drug Center, Denver Health and Hospital Authority, CO, USA

Thomas C. Arnold
Department of Emergency Medicine, Louisiana State University Health Sciences Center, Shreveport, LA

Richard B. Schwartz
Department of Emergency Medicine, Medical College of Georgia, Augusta, GA

David J. Vearrier
Department of Emergency Medicine, University of Mississippi Medical Center, Jackson, MS

Brian J. Wolk
Department of Emergency Medicine, Loma Linda University Medical Center, Loma Linda, CA

Tushar Gopalakrishna
Department of General Medicine, Father Muller Medical College Hospital, Mangalore, Karnataka, India

Department of Emergency Medicine, University of Florida College of Medicine, Jacksonville, FL

- Sophia S. Sheikh
- Dawn R. Sollee

Harish Kumar
Sardar Patel Medical College, Bikaner, Rajasthan, India; Email: drharishsgnr@gmail.com

Samuel J. Francis
Department of Emergency Medicine, Duke University, Durham, NC

Madhu Kumar
Mysore Medical College and Research Institute, Mysore, Karnataka, India

Sujoy Sarkar
Calcutta National Medical College, Kolkata, West Bengal, India

Adiel Aizenberg
Department of Emergency Medicine, College of Medicine University of Arizona, Tucson, AZ

Regan A. Baum
Department of Pharmacy, University of Kentucky HealthCare, Lexington, KY

Tamilarasu Kadhiravan
Jawaharlal Institute of Postgraduate Medical Education and Research, Puducherry, India

Narayanan Parameswaran
Jawaharlal Institute of Postgraduate Medical Education and Research, Puducherry, India

Nusrat Shafiq
Post Graduate Institute of Medical Education and research, Chandigarh, India

Deba Prasad
Post Graduate Institute of Medical Education and research, Chandigarh, India

Pannu Ashok Kumar
Post Graduate Institute of Medical Education and research, Chandigarh, India

Chandni Radhakrishnan
Government Medical College, Kozhikode, Kerala, India

Rojith Karandode Balakrishnan
Govt Medical College, Kozhikode, Kerala, India

Neeraj Manikath
Department Of Emergency Medicine, Government Medical College, Kozhikode, Kerala, India

Taylor Haston
Department of Emergency Medicine, Medical College of Georgia, Augusta, GA

John R. Hoskins
The Marcantonio-Hoskins Group, LLC

William Betts
William Betts III, LLC

Nick Cammack
Wellcome Trust

Lindsey Garver
US Army Medical Materiel Development Activity, Ft Detrick, MD

Ophirex, Inc., Corte Madera, 94925 CA, USA

- Brandi A. Ritter
- Jeffrey L. Owen
- Dana C. Miletic
- Suraj C. Ooman
- Nancy J. Koch
